# Endocrine therapy for metastatic solid pseudopapillary neoplasm of the pancreas: A case report

**DOI:** 10.3389/fonc.2022.970142

**Published:** 2022-09-13

**Authors:** Anna Kornietskaya, Sevindzh Evdokimova, Andrei Kachmazov, Alexander. Fedenko, Larisa Bolotina, Dmitriy Sidorov, Nadezhda Volchenko, Natalia Goeva, Anastasia Govaleshko, Andrey Kaprin

**Affiliations:** ^1^ P. Hertsen Moscow Oncology Research Institute – branch of the National Medical Research Radiological Centre of the Ministry of Health of the Russian Federation, Moscow, Russia; ^2^ City Clinical Oncological Hospital No1, Moscow, Russia; ^3^ Peoples’ Friendship University of Russia, Moscow, Russia

**Keywords:** solid pseudopapillary neoplasm (SPN), pancreas, tamoxifen, endocrine therapy, case report

## Abstract

Solid pseudopapillary neoplasm (SPN) of the pancreas is an extremely rare tumor, associated with favorable prognosis and long-term survival in patients with advanced disease. However, limited data exist on systemic therapy for such patients. Herein, we present a case of a young woman with a history of SPN, who progressed after multiple surgical resections and chemotherapy regimens. The immunohistochemistry (IHC) showed overexpression of estrogen receptors (ER) and progesterone receptors (PR) in tumor tissue. The patient started to receive tamoxifen and showed a durable response to endocrine therapy.

## Introduction

Solid pseudopapillary neoplasm (SPN) of the Pancreas, also known as Frantz’s tumor, is an exceedingly rare kind of neoplasm. It accounts for approximately 2% of all exocrine pancreatic malignant tumors, and 5% of cystic pancreatic neoplasms (1, 2). We searched PubMed for eligible case reports between January 2018 and August 2022 using key words: pseudopapillary neoplasm or Frantz tumor. From 520 articles, 512 cases include patients with confirmed diagnosis of SPN. Usually SPN is presented with a single node, however, multiple lesions in the pancreas are also described (3, 4). Moreover, there are reports of extrapancreatic SPNs, which may arise in the omentum, mesentery of the colon, retroperitoneally behind the head of the pancreas (5–7).

Available data shows that patients with SPN have a significantly better prognosis compared to ductal pancreatic adenocarcinoma. However, SPN is often diagnosed on locally advanced stage, which complicates possibility of radical surgical treatment and results in frequent recurrences (8).

The main strategy of SPN treatment is surgery (9). The overall survival and disease-free survival rates are strongly associated with the achievement of radical resection of tumor in patients with locally advanced disease and with distant metastases (2, 9, 10). According to literature, distant metastases occur in 7.7% of cases, while lymph node involvement is found in approximately 1.6% of cases (9).

There are alternative treatment options like systemic chemotherapy or radiotherapy when the process becomes unresectable (11–13). However, systemic cytotoxic treatment leads to limited results and objective responses are rarely achieved. Currently, there are no standard chemotherapy regimens for these patients.

Interestingly, this tumor appears more common in young women (14, 15). Jiali Wu et al. revealed the association between tumor size and menopausal status: SPNs were significantly bigger in premenopausal females. This indicates that female hormones may affect the growth of these neoplasms (16).

Another study has shown differences in SPN morphology depending on the sex of the patient (17). It included an equal ratio of male and female patients. The samples from women showed the typical features of SPNs (i.e., mostly consisting of cystic component, encapsulation by thick fibrous tissue, and ossification predominance). In contrast, tumors from men were shown to be prevalently occupied by solid components that lacked prominent pseudopapillary or pseudoglandular dystrophy.

In this point of view, investigation of possible endocrine therapy options is promising. We report a case of a young woman with metastatic SPN with positive estrogen and progesterone receptors in the tumor, who had progressed on multiple lines of chemotherapy, but maintained stable disease with tamoxifen treatment for a year.

## Case report

A 32-year-old-female presented to the local hospital with severe abdominal pain in 2011. CT (computed tomography) scan showed a round shaped hypodense mass in the pancreatic tail. Biopsy revealed solid pseudopapillary pancreatic tumor with foci of necrosis and hemorrhages (Picture 1 A-C). Distal pancreatectomy and splenectomy were performed. Microscopically, the tumor was represented by small monomorphic cells forming solid and pseudopapillary structures with poorly organized intercellular connections. Individual tumor cells are grouped around blood vessels, the nuclei are oriented oppositionally to the vessel lumen. The cell nuclei are round- or oval-shaped, with finely dispersed chromatin. The cytoplasm is eosinophilic, there are cells with foamy cytoplasm. The figures of mitoses are single in 10 fields of vision (x 40). The IHC staining was performed which revealed positivity for CD10, β-catenin, Synaptophysin (**Figure 3**). The diagnosis of SPN has been confirmed.

**Figure 1:**
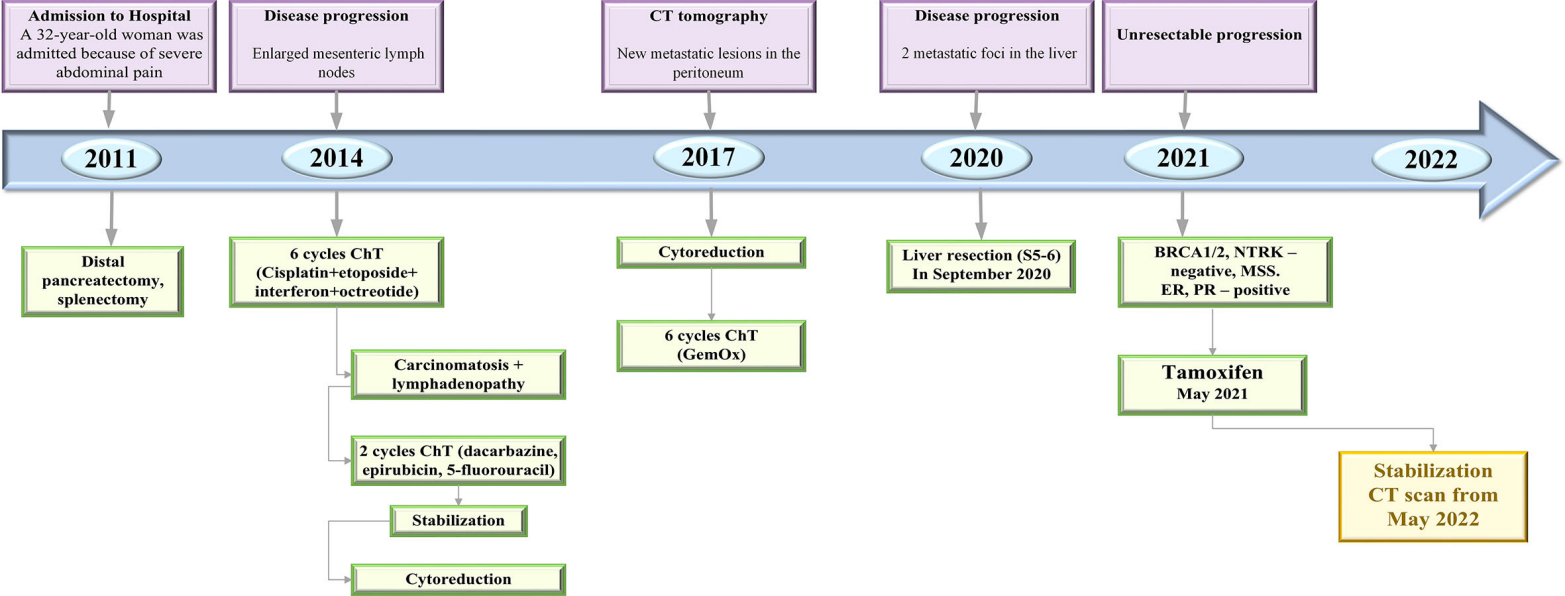
Timeline of treatment and disease characteristics. ChT, chemotherapy; CT, computed tomography; GemOx, gemcitabine oxaliplatin; ER, estrogen receptor; PR, progesterone receptor.

**Figure 2:**
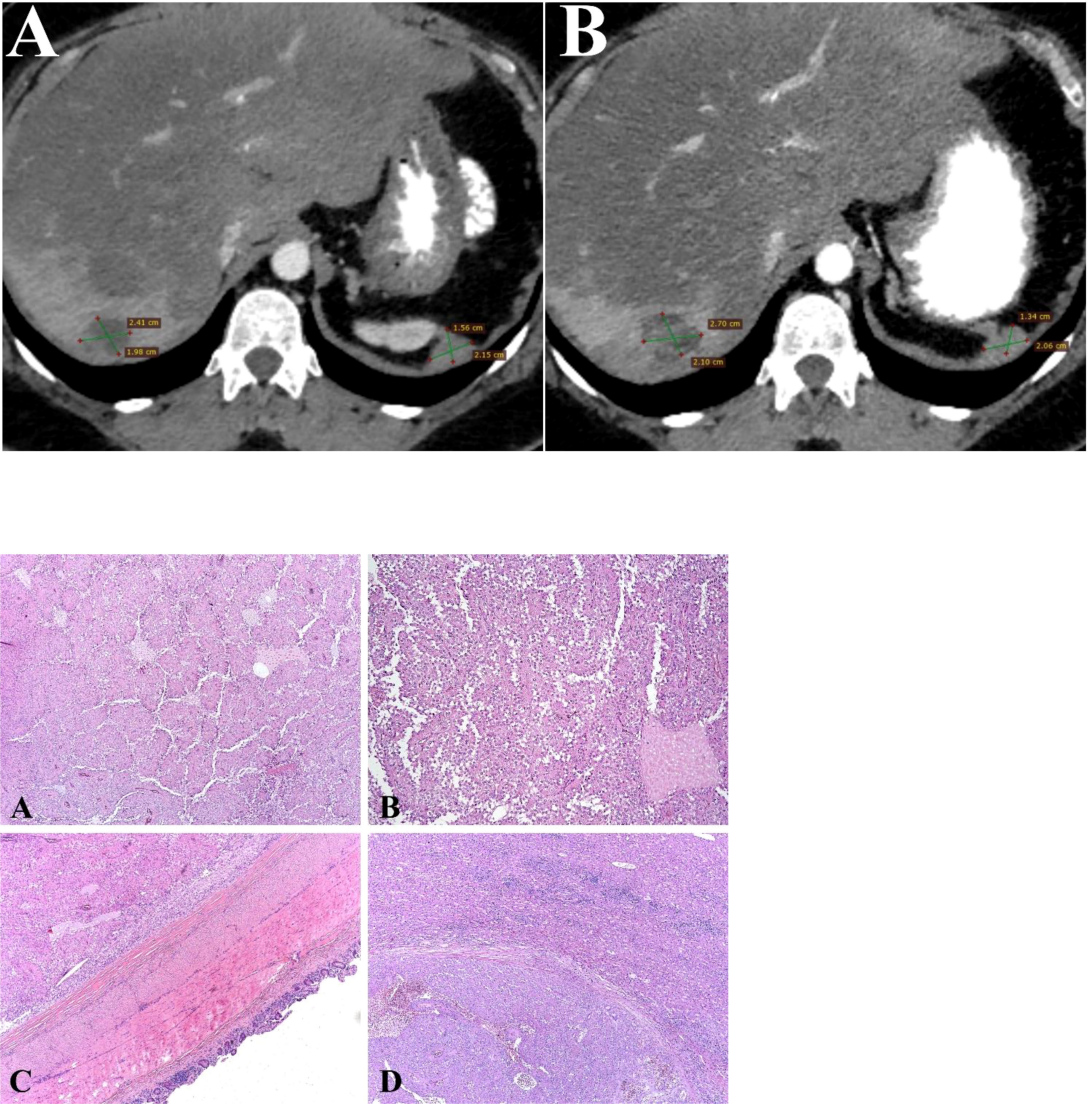
Contrast-enhanced CT scan image (Axial image). **(A)** Liver metastases from SPN of the pancreas in right lobe of liver at baseline examination. CT scan shows hematogenous dissemination of malignant nodules in the peritoneal space, retroperitoneal spaces at baseline examination. **(B)** Liver metastases from SPN of the pancreas in right lobe of liver after treatment. CT scan shows hematogenous dissemination of malignant nodules in the peritoneal space, retroperitoneal spaces after treatment. Picture 1 **(A, B)**. SPN of the Pancreas: The tumor is represented by monomorphic polygonal and rounded cells with the formation of capillary-like (pseudopapillary) structures formed as a result of violation of intercellular contacts. Tumor cells surround blood capillaries in the form of rosettes. (H&E, х20). **(C)**. SPN of the Pancreas: The tumor grows into the wall of the small intestine. (H&E, х20) **(D)**. SPN of the Pancreas: Tumor metastasis to liver tissue (H&E, х20).

Three years later, she developed enlarged lymph nodes in the mesentery and was treated with 6 cycles of cisplatin plus etoposide with combination of interferon and octreotide (**Figure 1**). Post-treatment abdomen CT showed continuing growth of mesenteric lymph nodes and small foci on the peritoneum. These findings were suggestive for peritoneal carcinomatosis. The chemotherapy regimen included itself dacarbazine, epirubicin, 5-fluorouracil. Some mild reduction in the tumor was indicated, whereafter cytoreduction surgery was done. In the first quarter of 2017, CT scan of the abdomen showed new metastatic lesions in the peritoneum. The patient underwent another surgical treatment with removal of metastatic foci of the abdominal cavity. Thereafter she was on 6 cycles of gemcitabine plus oxaliplatin (GemOx) until March 2018. A follow-up CT scan, which was performed 2 years after postoperative chemotherapy, revealed 2 lesions in the liver. Liver metastases were surgically removed. In May 2021, the progression became unresectable because of the occurrence of carcinomatosis in the abdominal cavity, pelvis, along the diaphragm and metastatic foci in the liver (**Figure 2A**).

**Figure 3:**
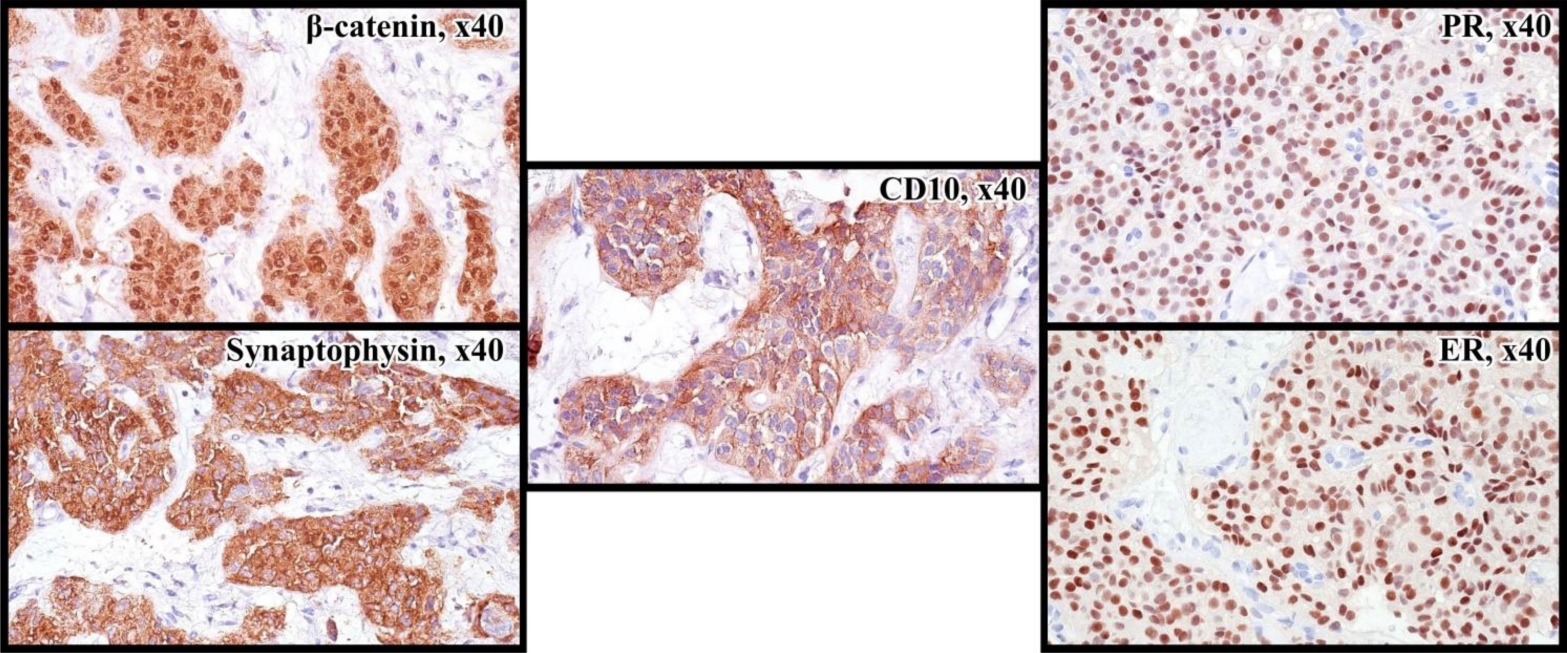
Immunoprofile of SPN: nuclear and cytoplasmic reaction with antibodies to β-catenin, cytoplasmic reaction with Synaptophysin, CD10, nuclear expression of ER and PR in 90% of tumor cells.

Due to lack of possible chemotherapy options, additional morphological examination showed tumor to be breast cancer gene 1/2 (BRCA 1/2), neurotrophic tyrosine receptor kinase (NTRK) – negative and microsatellite stability (MSS), which was analyzed by the immunohistochemistry (IHC). However, IHC analysis showed 7 by Allred score of estrogen receptors (ER) and progesterone receptors (PR) (**Figure 3**) in the tumor tissue, respectively. The patient has started to receive tamoxifen 20 mg per day without any associated drugs from May 2021. Since then CT scan from May 2022 showed durable stable effect (**Figure 2B**). This treatment is well tolerated without any significant adverse effects.

## Discussion

SPNs are rare pancreatic neoplasms, which usually are diagnosed on the locally advanced stages, with long-term survival rates after radical surgical resection. In the absence of metastases and a negative resection margin, surgical treatment provides a 97% of 5-year survival rate (18). However, even after radical resection of the tumor, 10–15% of patients develop local recurrence or distant metastases, mainly in the liver.

Given the low-grade malignancy, favorable prognosis, non-aggressive growth, the median overall survival is durable even with an unresectable disease. This could be confirmed by a literature report of a patient with an unresectable SPN, who survived for 25 years without treatment (18).

As for characteristics of SPN tissue and primary cell line, macroscopically, it is typically a single node, clearly outlined, often encapsulated. On the incision, the node tissue is of a soft consistency, light brown in color, often with the formation of cysts filled with dark brown contents.

Microscopically, the tumor is heterogeneous, represented by areas of solid, cystic and pseudopapillary structure of relatively monomorphic polygonal or rounded cells forming rosette-like structures around thin-walled blood vessels. Mitotic activity is usually low, does not exceed 10 mitoses in 10 visual fields (x40).

In immunohistochemical study, nuclear expression is characteristic β-catenin in tumor cells, in almost 100% of cases. Cyclin D1 expression is observed in 75% of cases. In addition, half of the observations recorded the expression of epithelial differentiation antigens, such as SC7, SC8, SC19, and a positive reaction with antibodies to Vimentin.

Differential diagnosis is performed with pancreatic acinocellular cancer and neuroendocrine tumors. Acinocellular cancer is characterized by the formation of acinar and glandular structures, granular cytoplasm of tumor cells, and lack of reaction with antibodies to β-catenin. Neuroendocrine tumors are represented by monomorphic cells with small nuclei and chromatin of the “salt and pepper” type. They express Chromogranin A and Synaptophysin.

Meanwhile, there is no data on effective systemic treatment for unresectable, recurrent, or metastatic disease. There are individual clinical cases that suggested the use of different chemotherapy regimens such as gemcitabine; oxaliplatin, leucovorin, fluorouracil (FOLFOX); irinotecan, leucovorin, fluorouracil (FOLFIRI), ifosfamide, cisplatin and etoposide (19–21). Moreover, there is data on the use of targeted drugs such as mTOR inhibitor Everolimus and multi-targeted receptor tyrosine kinase inhibitor Sunitinib (22, 23).

Hence, one of the key strategies of treatment of SPN with positive ER/PR may be using of hormone therapy. Tamoxifen is a selective estrogen receptor modulator. It is usually used for treatment of luminal breast cancer but also in patients with other tumors, which express hormone receptors such as ovarian cancer, desmoid tumors, and endometrial carcinoma. It is typically well tolerated by patients, with common side effects such as hot flashes, ocular disorders (including cataract, retinal thrombosis, retinopathy, vision color changes etc.), thrombolic events and uterine malignancies, occurring in less than 10% of cases.

Other hormone agents like aromatase inhibitors or fulvestrant with combination of ovarian suppression may also be considered as an alternative treatment method; however, there are no clinical cases on this topic.

Tognarini I et al. investigated the role of Tamoxifen on SPNs proliferation *in vitro*, where it has demonstrated substantial antiproliferative effect in cultured SPN cells with strong expression of ER (24). As for clinical practice, there are several reports of successful Tamoxifen treatment with accounts of a stable disease maintained for 12 years in a patient with unresectable local disease, as well as evidence of antiestrogen drug being effective in patient with liver metastasis (25, 26).

There are numerous studies, which report the expression of estrogen and progesterone (17, 27). The last one was presented in 79% of patients with SPN (28, 29). Despite this fact, the evidence of using endocrine therapy in real clinical practice is still very limited. Currently, there are no clinical studies on this issue, and the data is limited to several case reports (25, 26).

Considering the *in vitro* and pathology data that we have, it is necessary to determine the expression of estrogen and progesterone in SPN biopsy samples, in the process of clinical decision-making.

Taking into account the relatively favorable prognosis, low aggressiveness, as well as the presence of hormonal expression in more than 75% of cases, endocrine therapy with tamoxifen seems to be the valuable option of systemic treatment of patients with unresectable SPN of pancreas. It seems to be a preferred treatment strategy for patients with severe concomitant diseases, long relapse-free interval and in the absence of symptoms of the disease. Moreover, the quality of life of patients receiving hormone therapy is undoubtedly higher than that of those receiving cytostatic therapy.

## Conclusion

In summary, it is one of the few care reports of a durable stable response on tamoxifen in a patient with metastatic SPN. Our clinical case confirms the theory about possible using of hormone therapy in patients with exhausted treatment options. Additional studies of endocrine therapy are required for patients with metastatic or recurrent forms of SPN.

## Data availability statement

The original contributions presented in the study are included in the article/supplementary material. Further inquiries can be directed to the corresponding author.

## Ethics statement

The studies involving human participants were reviewed and approved by Ethics Committee of the National Medical Radiology Research Center (Moscow, Russia). The patients/participants provided their written informed consent to participate in this study.

## Author contributions

SE, AKor, AKac: Analysis of the results and writing of the manuscript. NG, NV: Molecular genetic and immunohistochemical studies. DS, AG: Patient treatment. AKap, AF, LB: Administration and management of workflows for the project. All authors contributed to the article and approved the submitted version.

## Conflict of interest

The authors declare that the research was conducted in the absence of any commercial or financial relationships that could be construed as a potential conflict of interest.

## Publisher’s note

All claims expressed in this article are solely those of the authors and do not necessarily represent those of their affiliated organizations, or those of the publisher, the editors and the reviewers. Any product that may be evaluated in this article, or claim that may be made by its manufacturer, is not guaranteed or endorsed by the publisher.
